# Supplementary material for: A popcorn-inspired strategy for compounding graphene@NiFe2O4 flexible films for strong electromagnetic interference shielding and absorption
Source: Nat Commun. 2024 Jun 28;15:5486. doi: 10.1038/s41467-024-49498-1 (PMC11213894; doi:10.1038/s41467-024-49498-1)
Supplement: Supplementary file 1 — Supplementary Information [file 41467_2024_49498_MOESM1_ESM.docx]

**Supplementary Information for A “popcorn-making-mimic” strategy for compounding graphene@NiFe_2_O_4_ flexible films for strong electromagnetic interference shielding and absorption**

Mingjie Liu^1^†, Zhiyuan Wang^1^†, Zhaoqiang Song^2,3^†, Fangcheng Wang^1,4^, Guangyao Zhao^1^, Haojie Zhu^1^, Zhuofei Jia^1^, Zhenbin Guo^2^*, Feiyu Kang^1^ and Cheng Yang^1^*

^1^Institute of Materials Research, Tsinghua Shenzhen International Graduate School, Tsinghua University, Shenzhen 518055, China

^2^Institute of Semiconductor Manufacturing Research, Shenzhen University, Shenzhen 518060, China

^3^Department of Materials Science and Engineering, University of Pennsylvania, Philadelphia, PA, USA

^4^Shenzhen Institute of Advanced Electronic Materials, Shenzhen Institute of Advanced Technology Chinese Academy of Sciences, Shenzhen, 518055, China

*Corresponding author. Email: [yang.cheng@sz.tsinghua.edu.cn](mailto:yang.cheng@sz.tsinghua.edu.cn); [guozb@szu.edu.cn](mailto:guozb@szu.edu.cn)

†These authors contributed equally to this work

**This PDF file includes:**

Supplementary Figure 1–17

Supplementary Tables 1–5

Supplementary Discussion (Figure 8, Figure 16, Table 2)

References


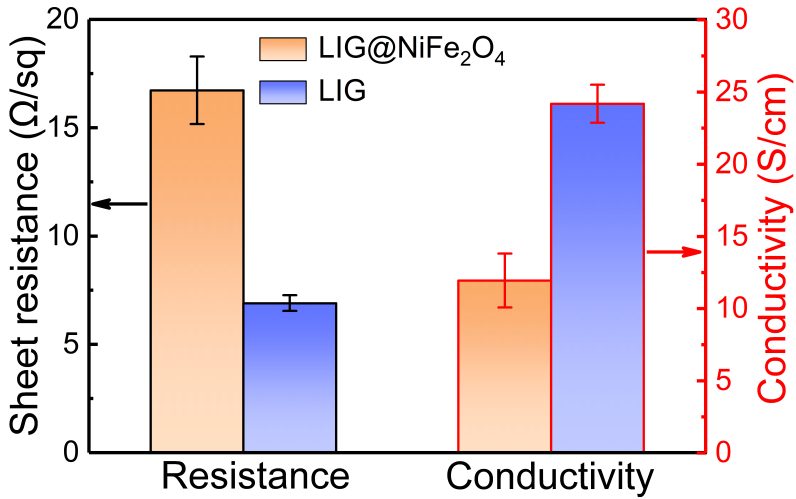


**Supplementary Figure 1.** Comparison of resistance and conductivity of LIG@NiFe_2_O_4_ and LIG. The error bars are derived from calculating the standard deviation of five samples.


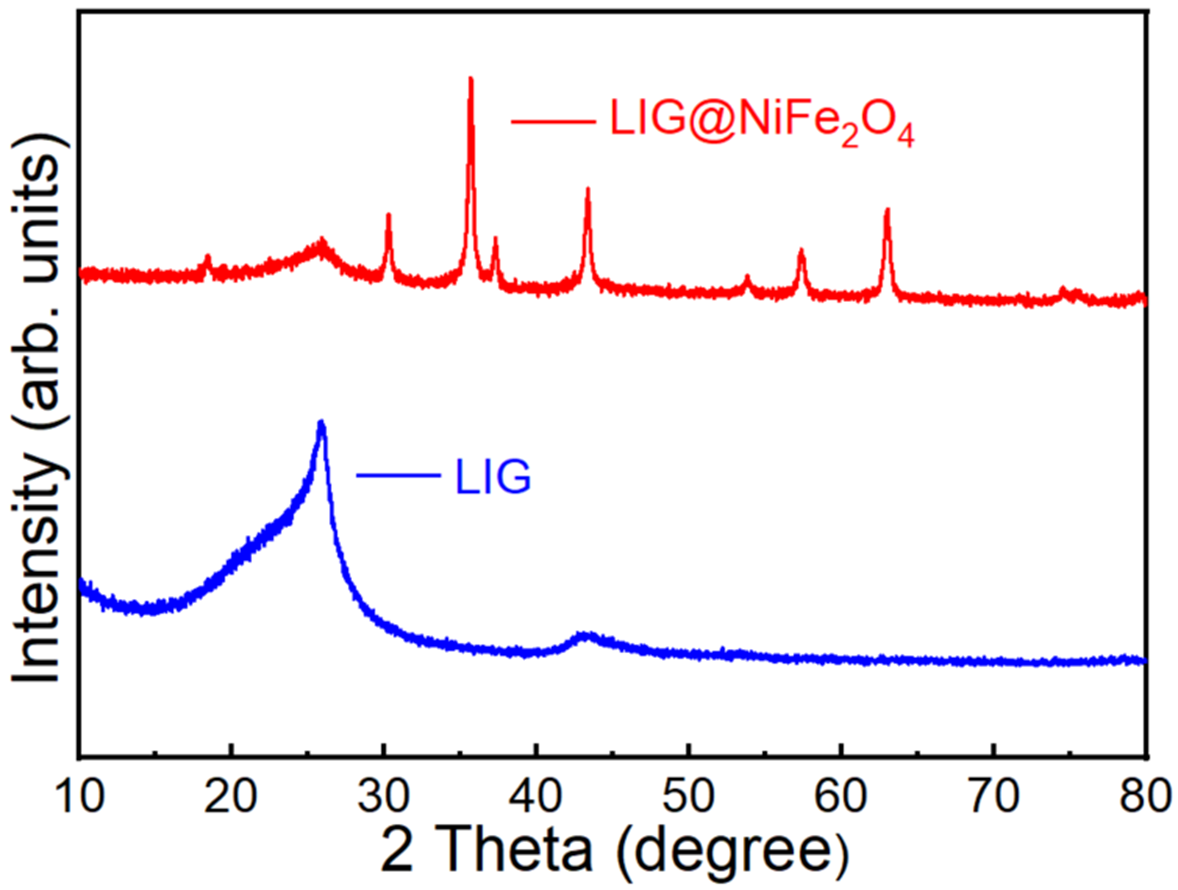


**Supplementary Figure 2.** XRD patterns of LIG@NiFe_2_O_4_ and LIG.


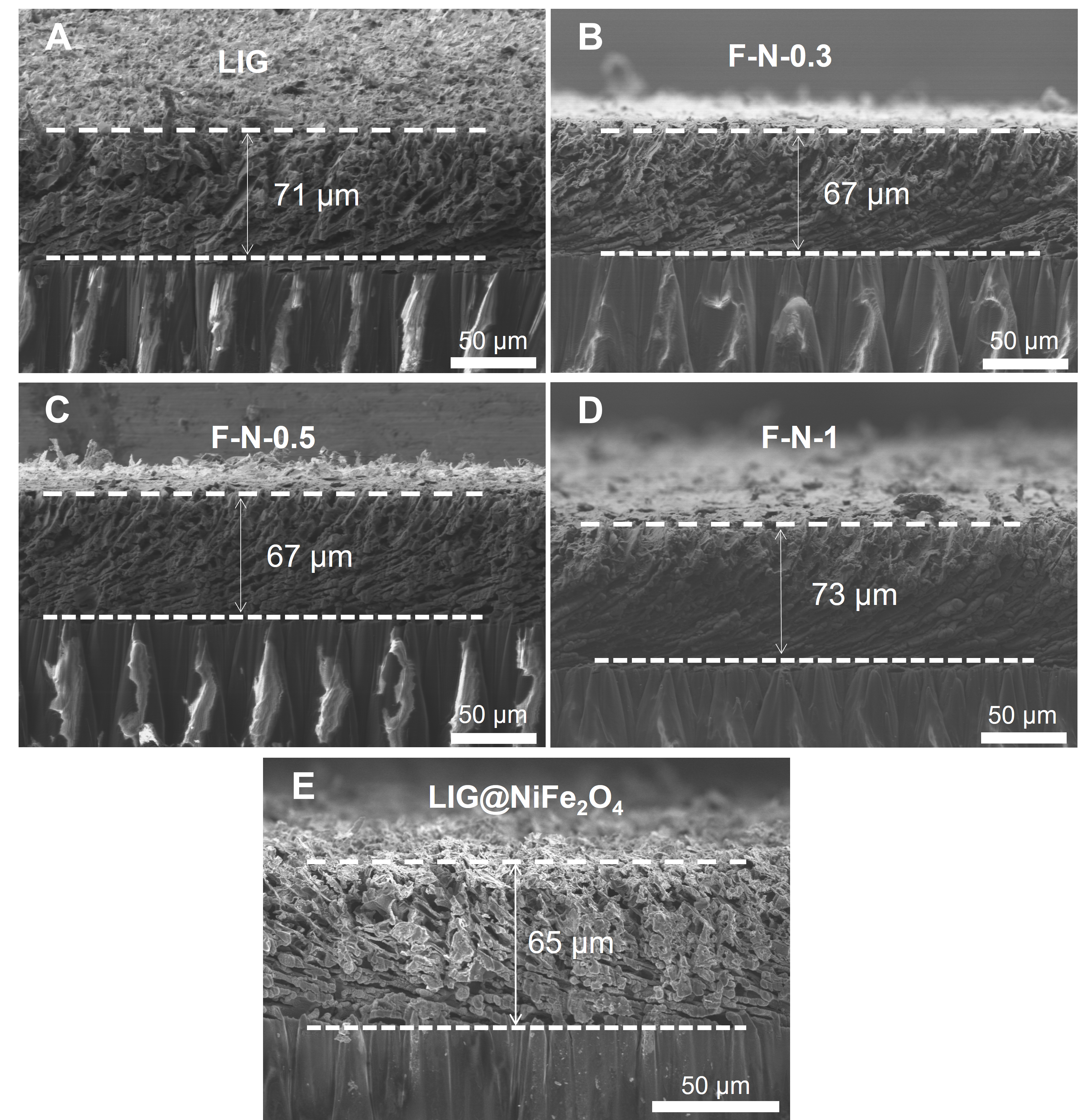


**Supplementary Figure 3.** Cross-sectional SEM images of LIG, LIG@NiFe_2_O_4_ and different rGO/LIG@NiFe_2_O_4_ samples


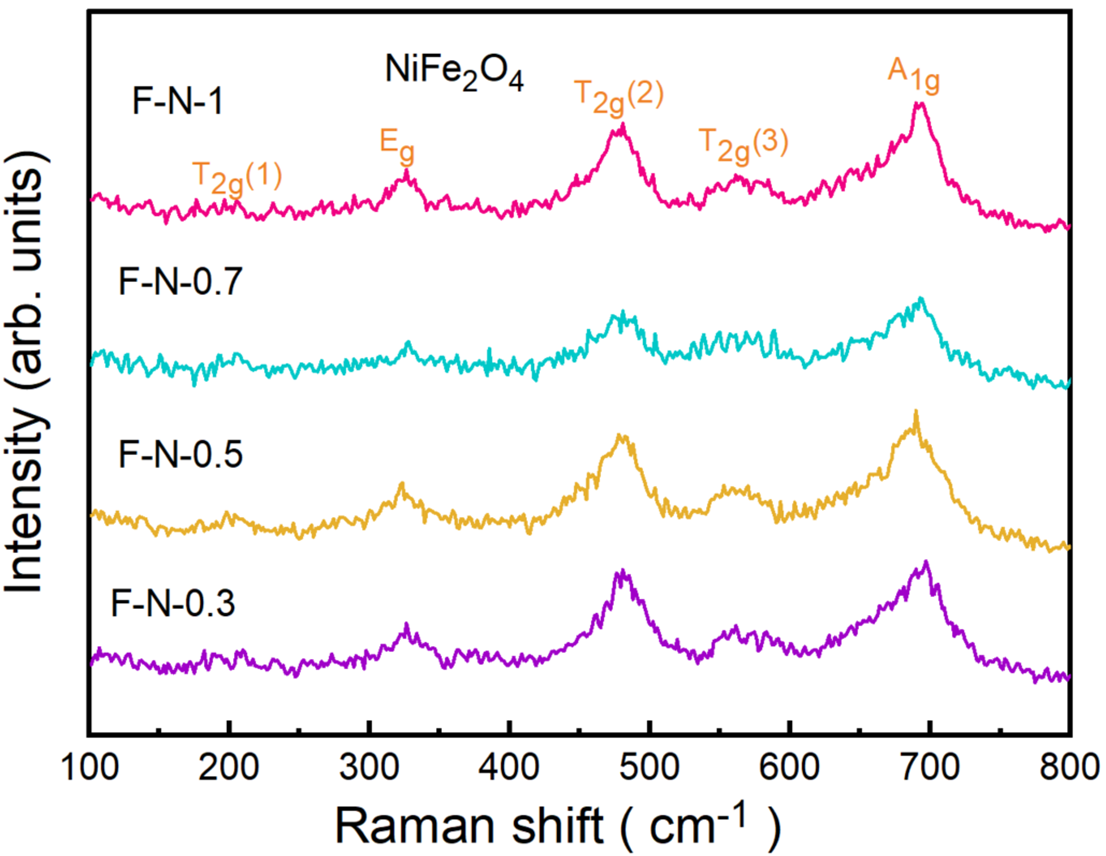


**Supplementary Figure 4.** Raman spectra of the NiFe_2_O_4_ nanoparticles.


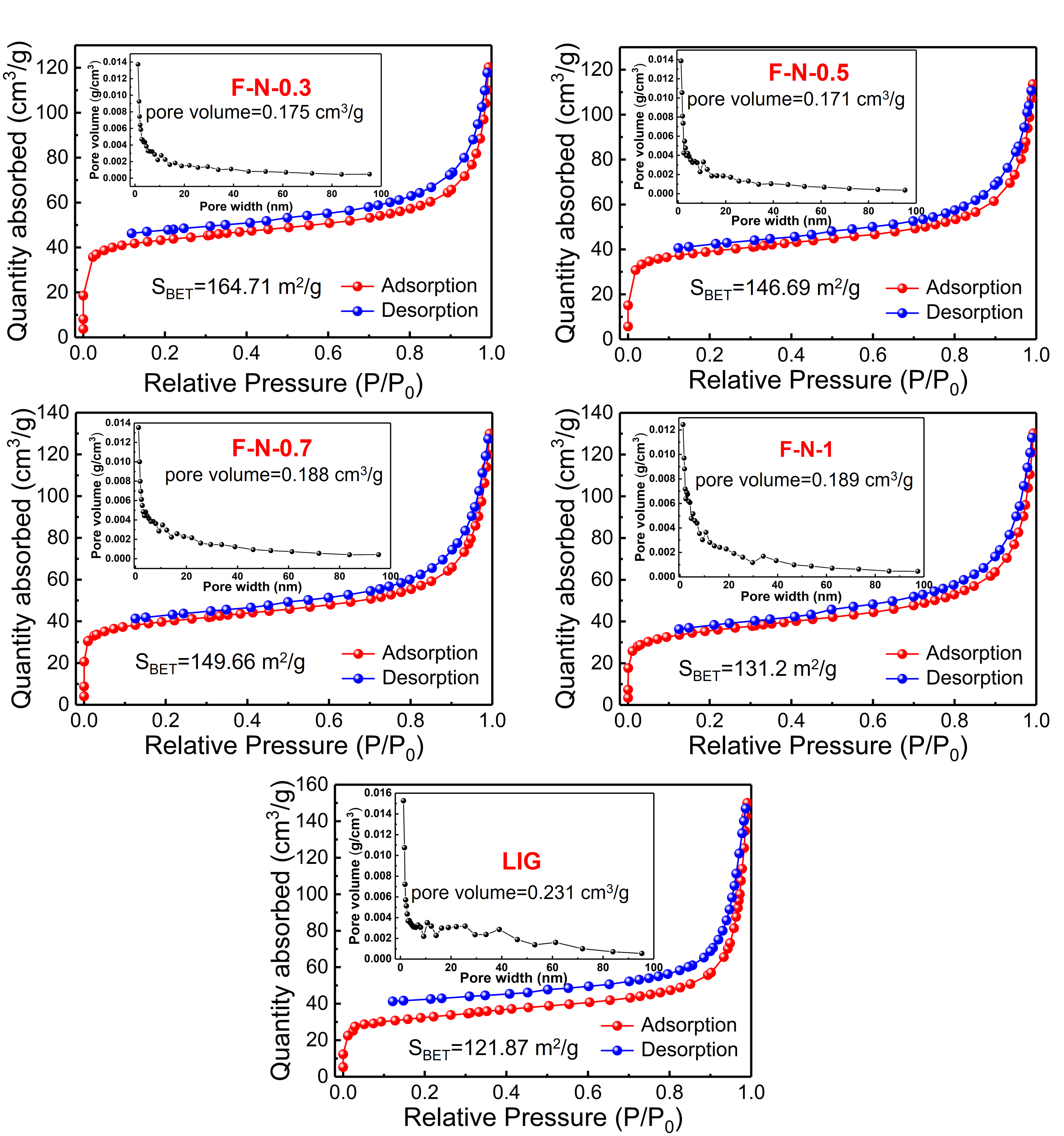


**Supplementary Figure 5.** Nitrogen adsorption and desorption curves of LIG and different rGO/LIG@NiFe_2_O_4_ samples.


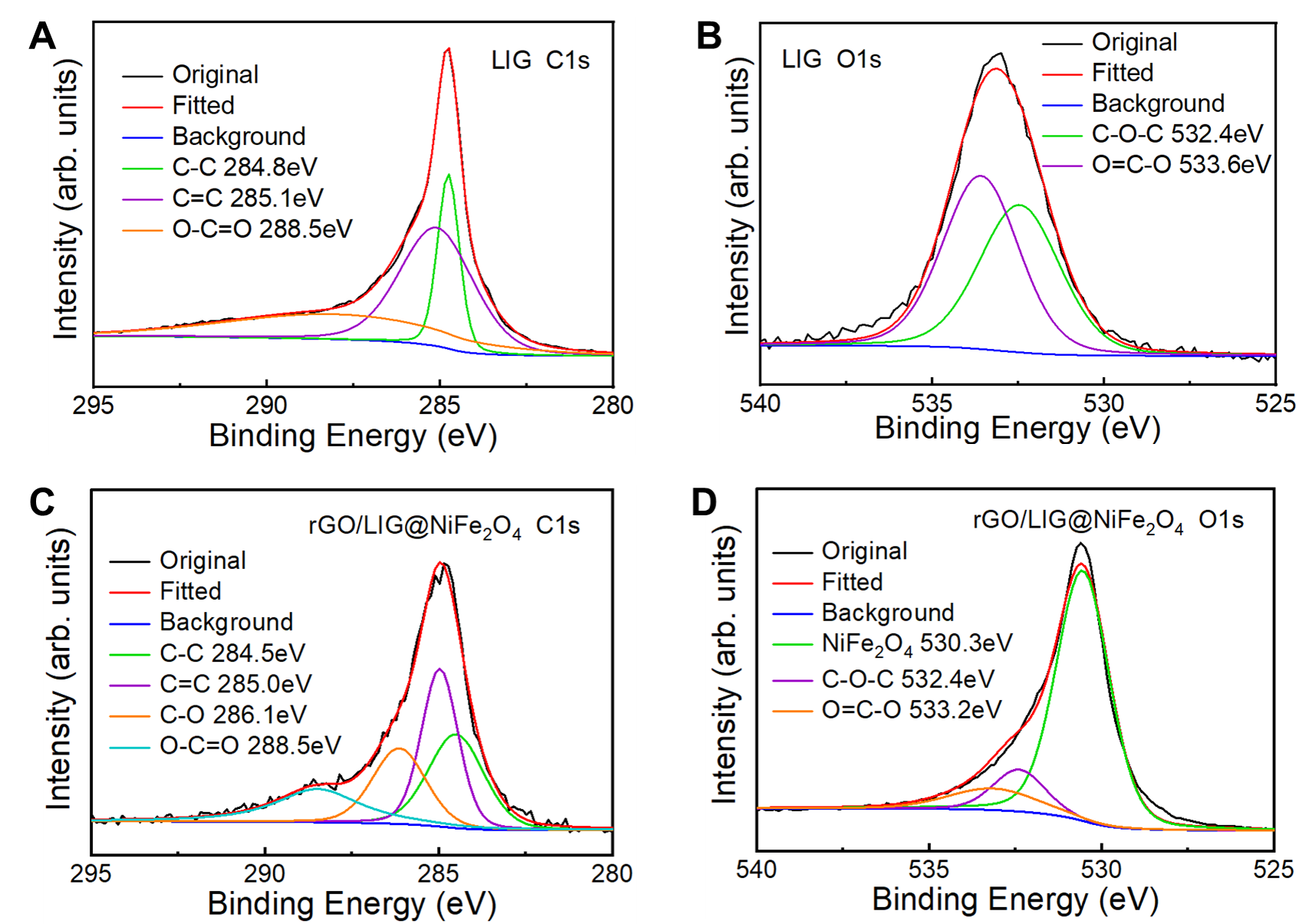


**Supplementary Figure 6.** XPS of LIG (A) C1s (B) O1s and rGO/LIG@NiFe_2_O_4_ (C) C1s (D) O1s.


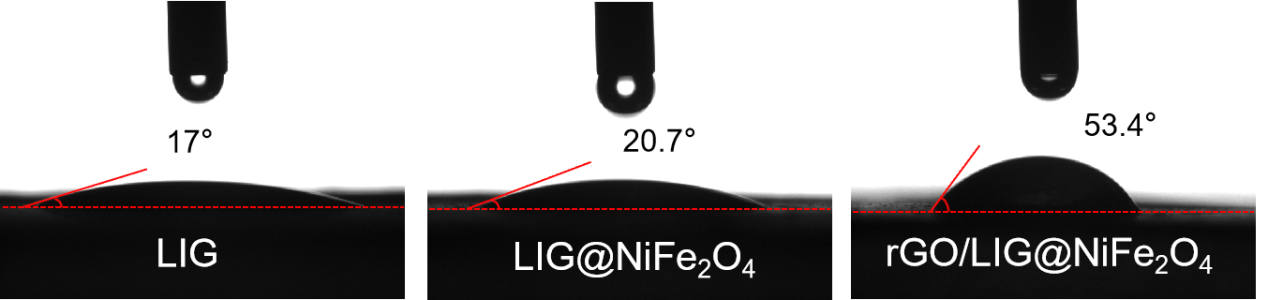
**Supplementary Figure 7.** A comparison of contact angle characterization results of the laser induced graphene (LIG), LIG@NiFe_2_O_4_ and rGO/LIG@NiFe_2_O_4_ (F-N-0.7).

**
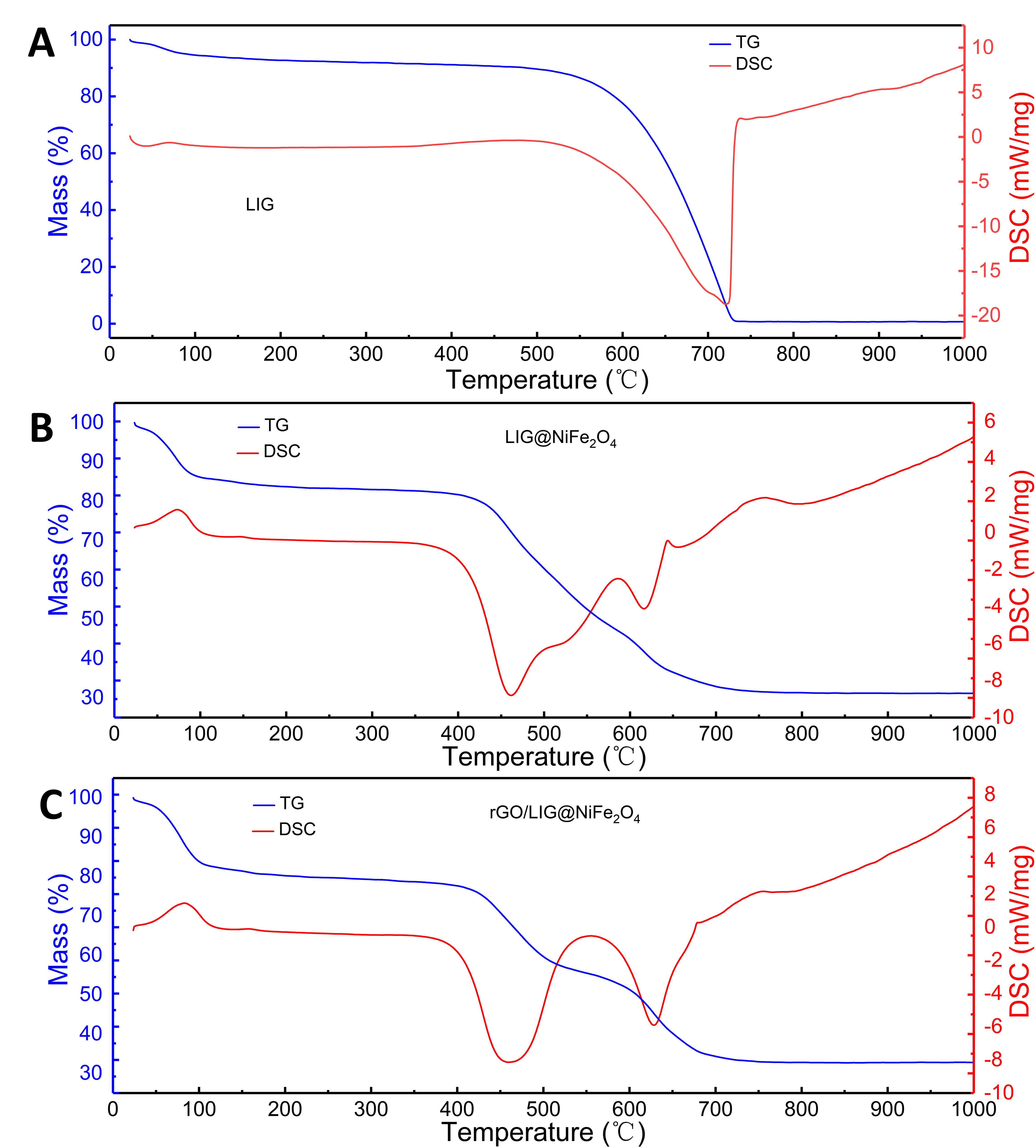
**

**Supplementary Figure 8.** (A-C) TG and DSC curves of LIG, LIG@NiFe_2_O_4_ and rGO/LIG@NiFe_2_O_4_ (F-N-0.7) (specific test conditions are heating from room temperature to 1000 ℃ at 10 ℃/min under ambient atmosphere)

In order to understand the thermal stability of LIG and LIG loaded with magnetic metal oxide nanoparticles as well as the influence of different loading levels of nanoparticles on the thermal decomposition process of LIG in air, we tested and analyzed the TGA-DSC curves of LIG. It can be seen from the TG and DSC curves of LIG in Supplementary Fig. 8A that its mass change at 0-100℃ is related to the decomposition and vaporization of various unstable functional groups of graphene, as well as the loss of residual (or absorbed) solvents. The rapid decline of its mass from 500 to 730℃ is caused by the intense combustion of LIG in the air, and it can be seen from the DSC curve as a strog exothermic peak, which is consistent with the situation reported in literature^3^. It can be seen from the TG and DSC curves of LIG@NiFe_2_O_4_ in Supplementary Fig. 8B that its mass change at below 100℃ is related to the loss of moisture adsorbed by LIG@NiFe_2_O_4_ composite film. The disintegration temperature of LIG was significantly down-shifted, indicating that the loading of NiFe_2_O_4_ nanoparticles accelerates this process. The heat absorption peak near 760℃ is related to the high-temperature oxidation of the nanoparticles, with valence states probably reduced during the LIG disintegration and then re-oxidized at this temperature range^4^. The TG-DSC curve of the composite film with rGO lid (Supplementary Fig. 8C) is similar to that of Supplementary Fig. 8B, because both samples contain NiFe_2_O_4_ nanoparticles, but with presence of the rGO lid, the LIG's burning becomes sluggish. To note, a broad exothermic peak appears at 460℃ on the left side of the DSC curve in Supplementary Fig. 8C. We believe that a reasonable explanation is that NiFe_2_O_4_ nanoparticles are evenly distributed, so the catalytically accelerate the disintegration process of LIG^5,6^.

**
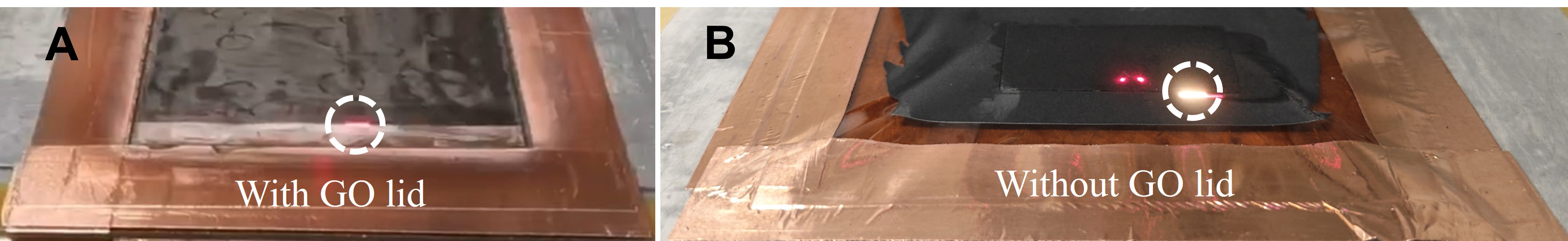
**

**Supplementary Figure 9.** (A-B) Photographic images of the laser preparation process of rGO/LIG@NiFe_2_O_4_ (F-N-0.7) and LIG@NiFe_2_O_4_. Both conditions used the same laser processing parameters, specifically: a scanning speed of 250 mm s^-1^, a scanning pitch of 0.005 mm, and a power percentage of 6% (The power was 2.43 W).

(Note: The two red dots in Supplementary Figure 9B are laser positioning marks, which have no effect on the experimental results)


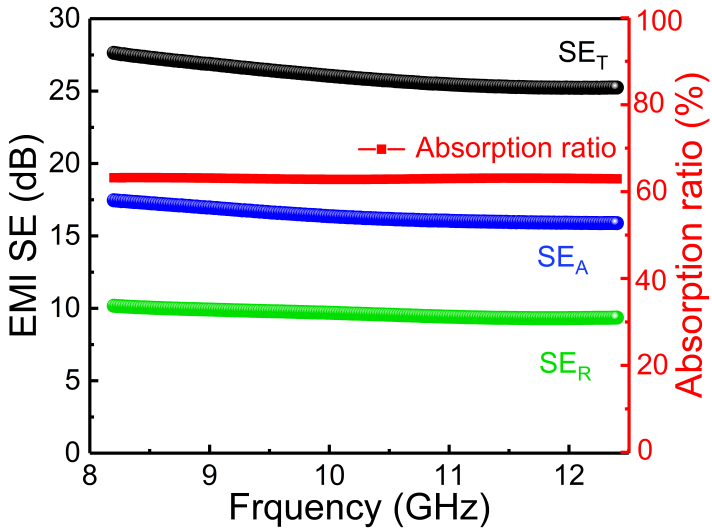


**Supplementary Figure 10.** EMI SE diagram of LIG@NiFe_2_O_4_.


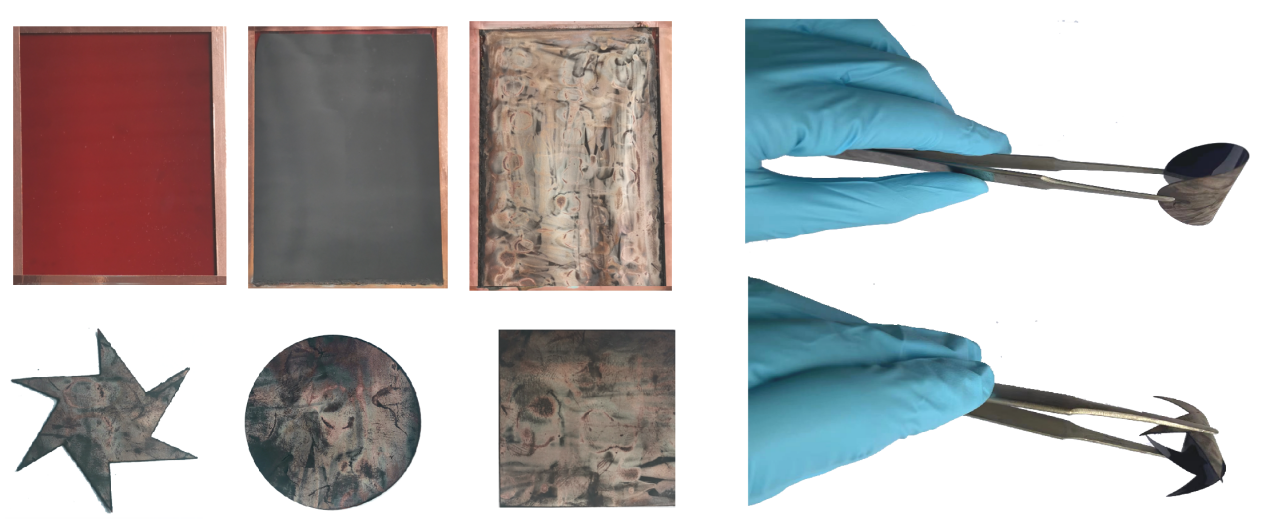


**Supplementary Figure 11.** (A) PI (Polyimide), LIG (Laser induced graphene) and rGO/LIG@NiFe_2_O_4_ physical photos. (B) Bending diagram for rGO/LIG@NiFe_2_O_4_ composite films. (C) Diagram of arbitrary cutting of rGO/LIG@NiFe_2_O_4_ composite film.


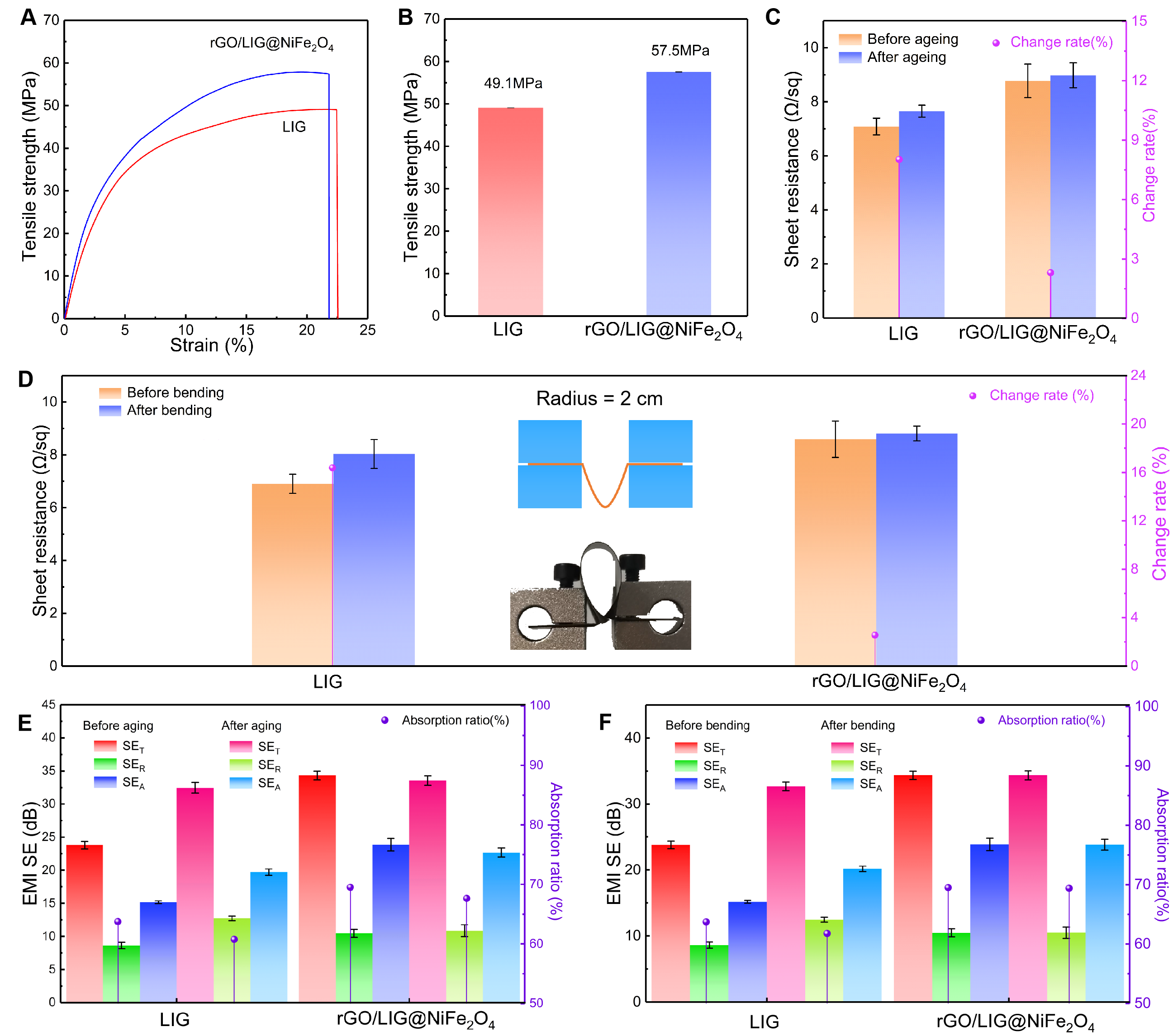


**Supplementary Figure 12.** Mechanical and stability performance characterizations: (A-B) Stress-strain curve and tensile strength of LIG and rGO/LIG@NiFe_2_O_4_ (F-N-0.7) composite film. (C-D) Square resistance and electrical conductivity of LIG and rGO/LIG@NiFe_2_O_4_ (F-N-0.7) composite films before and after bending and aging. (E-F) EMI shielding effectiveness of LIG and rGO/LIG@NiFe_2_O_4_ (F-N-0.7) composite films before and after bending and aging. The error bars are derived from calculating the standard deviation of five samples.


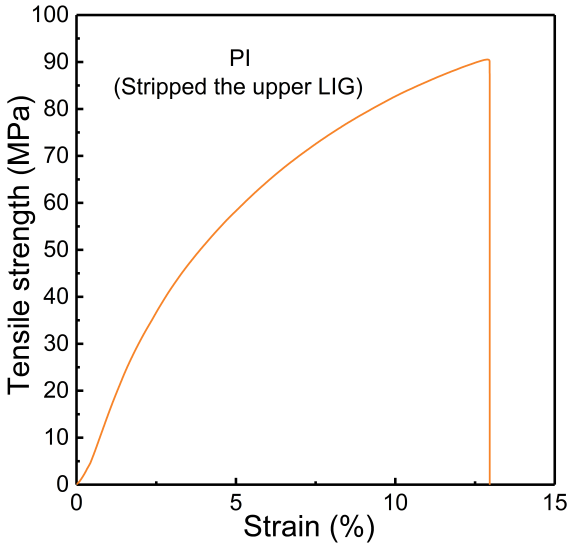


**Supplementary Figure 13.** The stress-strain curve of PI with the upper LIG stripped off.


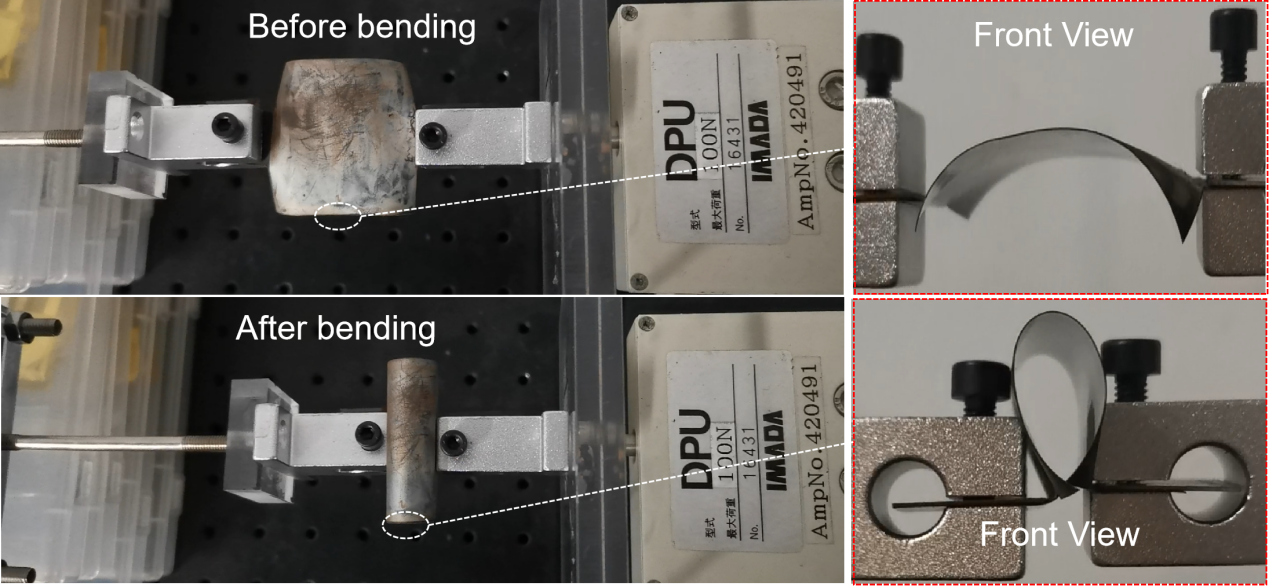


**Supplementary Figure 14.** Schematic diagram of mechanical bending of the rGO**/**LIG@NiFe_2_O_4_composite film.


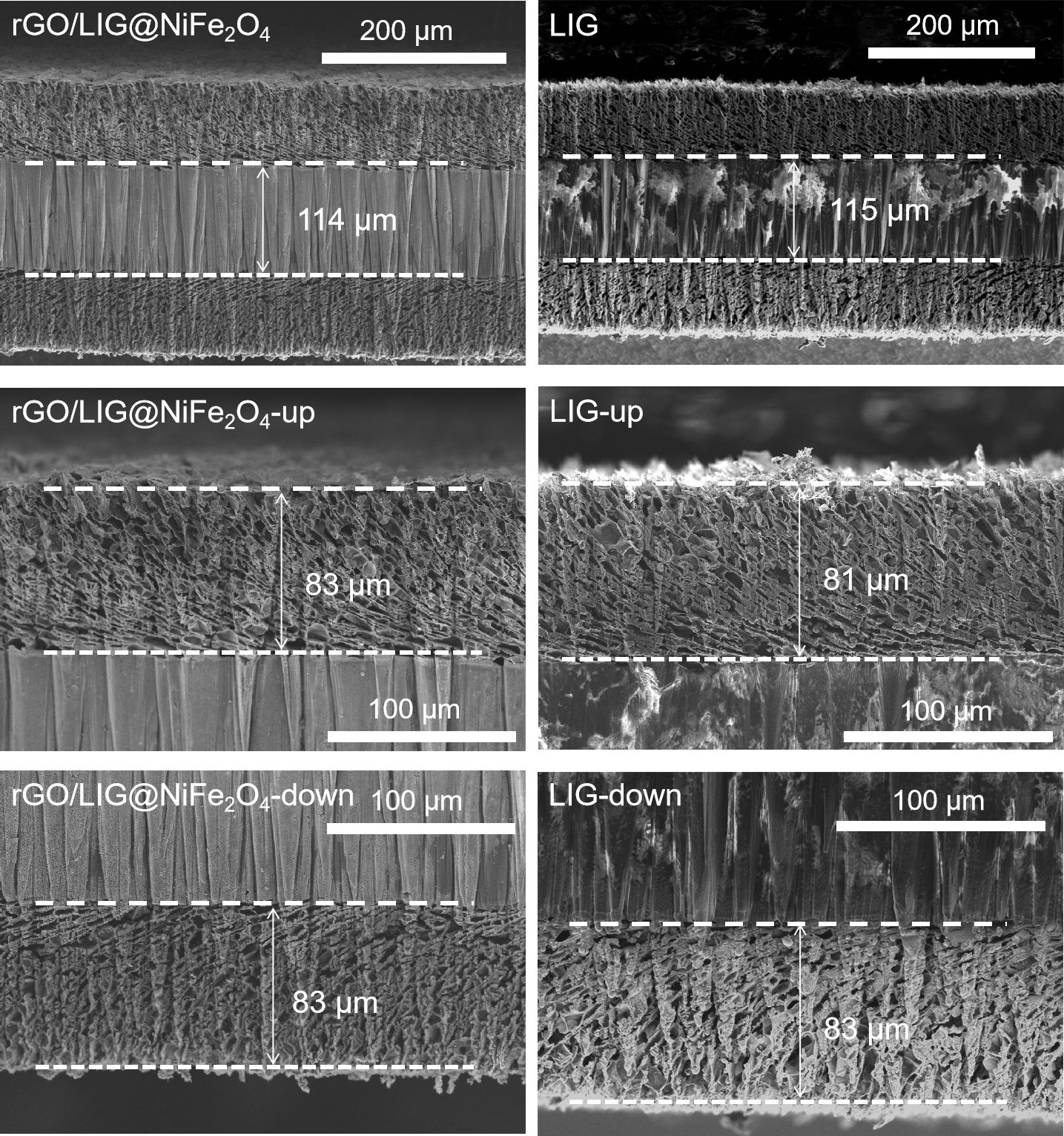


**Supplementary Figure 15.** Cross-sectional SEM images of double-sided LIG and rGO/LIG@NiFe_2_O_4_ (F-N-0.7) samples.


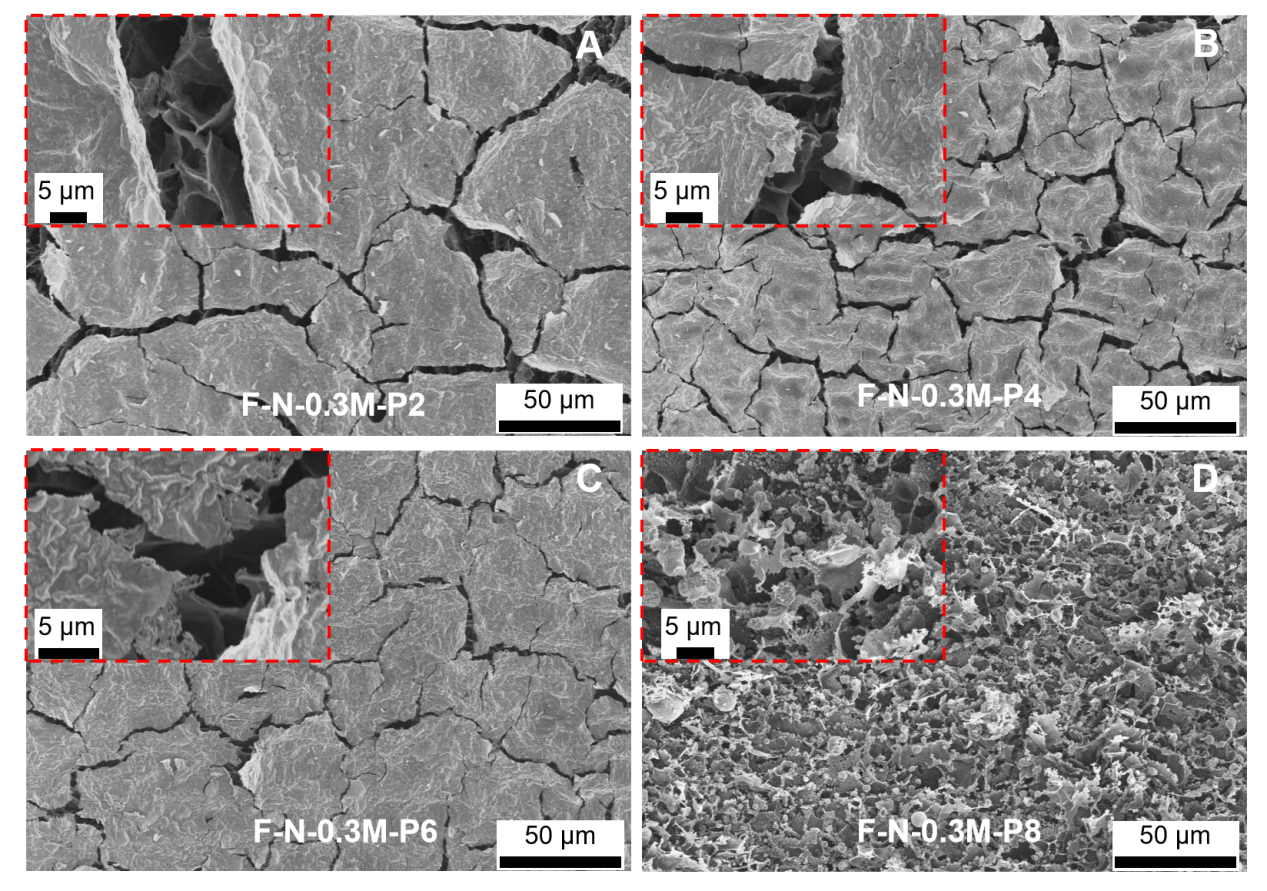


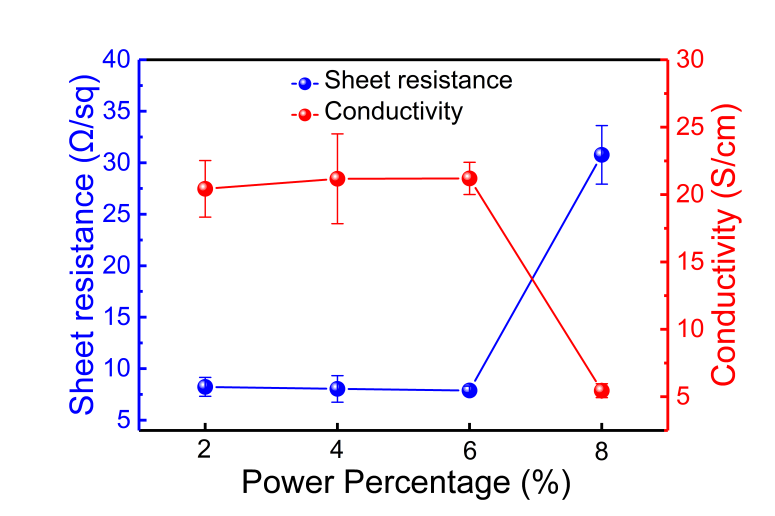


**E**

**Supplementary Figure 16.** (A-D) SEM images and (E) conductivity variation images of rGO/LIG@NiFe_2_O_4_ samples under different laser power percentages. (Note: xM in F-N-xM-Py represents the precursor salt concentration and Py represents the percentage of laser power) The error bars are derived from calculating the standard deviation of six samples

Given the important influence of laser power on the reduction of GO and the generation and loading of nanomaterials, this variable has been carefully studied and determined. It can be seen (Supplementary Fig. 16A, B, C, D and E) that different laser power outputs can lead to different overall morphology and conductivity of rGO/LIG@NiFe_2_O_4_. As marked in SEM plots (Supplementary Fig. 16A, B, C and E), when the laser power output increases from 2% to 6%, the conductivity of rGO/LIG@NiFe_2_O_4_ gradually increased, and rGO remained flat on the surface of the LIG, protecting the integrity of LIG. In addition, the surface of rGO showed a large number of chaps due to the escape of gases generated during the preparation of the rGO/LIG@NiFe_2_O_4_ composite film. Since GO is almost insulating (5×10^-6^ S∙cm^-1^ and 4×10^-3^ S∙cm^-1^)^1,2^, the overall high conductivity of rGO/LIG@NiFe_2_O_4_ can indicate that GO is successfully transformed into rGO. Compared with LIG, the electric conductivity of rGO/LIG@NiFe_2_O_4_ is slightly lower, which may be caused by the loading of NiFe_2_O_4_ on LIG with relatively poor electric conductivity. When the laser energy increased to 8%, the surface GO was burned and the structure of the internal LIG was also destroyed. The conductivity of rGO/LIG@NiFe_2_O_4_ dropped sharply compared to LIG. This indicates that a power percentage of 6% (4.22 W) is more favorable for the synthesis of the structure of the material and the reduction of GO. Ultimately, the structural integrity of LIG can be achieved to ensure that the conductivity is not greatly affected, while achieving uniform generation and loading of metal oxide nanoparticles onto the LIG and GO.

*
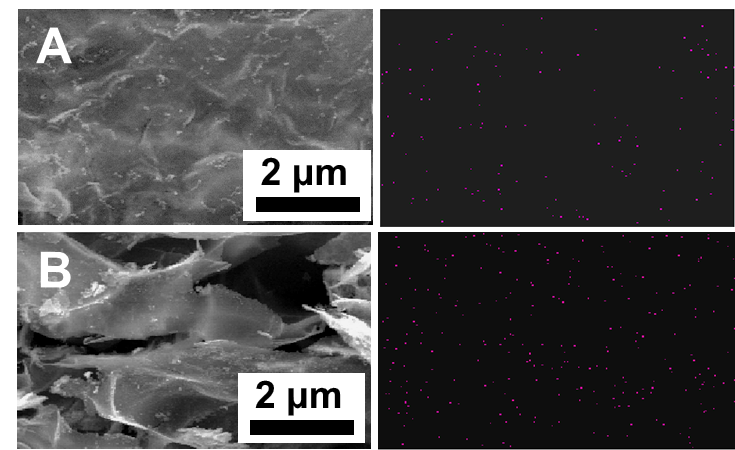
*

**Supplementary Figure 17.** SEM images and corresponding (Ni) EDX mapping results of rGO and LIG. (A) Top-view from the rGO lid surface. (B) Cross sectional-view of LIG.

**Supplementary Table 1.** Thickness, density and volumetric loading of NiFe_2_O_4_ for LIG and different rGO/LIG@NiFe_2_O_4_ samples

| **Sample** | **NiFe_2_O_4_ loading (g/cm^3^)** | **Thickness (µm)** | **Density of LIG**  **(g/cm^3^)** | **EMI SE**  **(dB)** |
| --- | --- | --- | --- | --- |
| LIG | 0 | 71 | 0.26 | 25.2 |
| rGO/LIG@NiFe_2_O_4_ (F-N-0.3) | 0.15 | 51 | 0.26 | 29.5 |
| (F-N-0.5) | 0.17 | 67 | 0.26 | 34.3 |
| (F-N-0.7) | 0.21 | 70 | 0.26 | 36.0 |
| (F-N-1) | 0.38 | 73 | 0.26 | 33.3 |
| double-layer LIG | 0 | 164 | 0.26 | 36.1 |
| double-layer rGO/LIG@NiFe_2_O_4_  (F-N-0.7) | 0.25 | 166 | 0.26 | 50.9 |

**Supplementary Table 2.** Masses of rGO, rGO/LIG@NiFe_2_O_4_ (F-N-0.7) and LIG@NiFe_2_O_4_ with a fixed area (40 x 40 mm)

| **Sample** | **Area (mm^2^)** | **Mass (mg)** | **Thickness (µm)** |
| --- | --- | --- | --- |
| LIG | 40 x 40 | 29.20 | 71 |
| rGO/LIG@NiFe_2_O_4_ (F-N-0.7) | 40 x 40 | 48.80 | 70 |
| rGO | 40 x 40 | 7.40 | 2.01-2.20 |
| LIG@NiFe_2_O_4_ | 40 x 40 | 37.55 | 65 |

We calculated the masses of rGO, LIG, rGO/LIG@NiFe_2_O_4_ (F-N-0.7), and LIG@NiFe_2_O_4_ with a fixed area (40 x 40 mm). The mass of the LIG was obtained by subtracting the mass of the substrate PI. To obtain the mass of the surface layer of rGO, we first applied an aqueous phase dispersion of GO (2 g of GO dispersed in 10 g of water, 300 µL drip coating) to the LIG, and then processed it by laser to obtain the rGO covering the LIG. Finally, the mass of rGO was obtained by subtracting the mass of the substrate and LIG. In addition, we calculated the theoretical thickness of the surface rGO lid to be 2.01-2.20 μm based on the previously reported density of graphene (2.1-2.3 g/cm^3^). It is remarkable that the mass of rGO/LIG@NiFe_2_O_4_(F-N-0.7) minus that of the surface rGO is 3.85 mg larger than the mass of LIG@NiFe_2_O_4_, which indicates that with the GO protection the mass retention is improved over 10% of the original weight after the laser treatment, which displays a significant difference.

**Supplementary Table 3.** Parameters for finite element analysis

| **Symbols** | **Meaning** | **Value** | **Ref** |
| --- | --- | --- | --- |
| $E$ | Elastic modulus of rGO | 2.0 TPa | Ref^1^ |
| $\nu$ | Poisson’s ratio of rGO | 0.18 | Ref ^2^ |
| $t$ | Thickness of rGO | 1 $\mu m$ | This work |
| $\rho$ | Density of rGO | 2.267 g/cm^3^ | Ref ^3^ |
| $\sigma_{th}$ | Strength of rGO | 130 GPa | Ref ^4^ |
| $k$ | Thermal conductivity of rGO | 4000 $W\cdot m^{-1}K^{-1}$ | Ref ^5^ |
| $\gamma$ | Emissivity of rGO | 0.025 | Ref ^6^ |
| $\alpha$ | Coefficient of thermal expansion (in-plane) of rGO | -2 x 10^-6^ K^-1^ | Ref ^4^ |
| $\alpha$ | Coefficient of thermal expansion (out-of-plane) of rGO | 6 x 10^-6^ K^-1^ | Ref ^4^ |
| $R_{c}$ | Reflection coefficient of rGO | 0.1 | Ref ^7^ |
| $r_{c}$ | Radius of laser spot | 25 $\mu m$ | This work |
| $c_{p1}$ | Heat capacity of rGO | 1 $J/(kg\cdot K)$ | Ref ^8^ |
| $k_{s}$ | Thermal conductivity of substrate | 10 $W\cdot m^{-1}K^{-1}$ | This work |
| $c_{ps}$ | Heat capacity of substrate | 700 $J/(kg\cdot K)$ | This work |
| $k_{p}$ | Thermal conductivity of NiFe_2_O_4_ | 50 $W\cdot m^{-1}K^{-1}$ | This work |
| $c_{p2}$ | Heat capacity of NiFe_2_O_4_ | 700 $J/(kg\cdot K)$ | This work |

**Supplementary Table 4.** Detailed parameters and references for each dimension of the six-dimensional radar plot

| **Filler** | **Thickness**  **(µm)** | **Flexibliity** | **Density**  **(g/cm^3^)** | **EMI SE**  **(dB)** | **SSE**  **(dB*cm^3^/g)** | **SSE/t**  **(dB*cm^2^/g)** | **Ref** |
| --- | --- | --- | --- | --- | --- | --- | --- |
| Graphene/Fe_3_O_4_ | 300 | 1 | 0.77 | 24 | 31.17 | 1038.96 | ^7^ |
| Cu foil | 10 | 3 | 8.97 | 70 | 7.80 | 7803.79 | ^8^ |
| SWCNT/MWCNT film | 130 | 3 | 0.85 | 65 | 76.83 | 5910.17 | ^9^ |
| MXene/ANF | 20 | 3 | 1.25 | 28 | 22.4 | 11200 | ^10^ |
| LIG | 71 | 4 | 0.26 | 25.2 | 102.44 | 14428.03 | This  work |
| (F-N-0.7)  rGO/LIG@NiFe_2_O_4_ | 70 | 4 | 0.26 | 36.1 | 146.34 | 20905.92 |  |
| double-side  rGO/LIG@NiFe_2_O_4_  (F-N-0.7) | 166 | 4 | 0.26 | 50.9 | 206.91 | 12464.49 |  |

(Notes on flexibility properties: The number of bends not indicated in the literature is marked as number 1, the number of bends illustrated with a bending schematic but not detailed is marked as number 2, the number of bends between 5,000 and 10,000 is marked as number 3 and the number of bends greater than 10,000 revolutions is marked as number 4)

**Supplementary Table 5.** Detailed parameters and references for the comprehensive performance comparison chart (thickness, EMI shielding performance and absorption rate)

| **Materials** | **Thickness**  **(µm)** | **Density**  **(g cm^3^)** | **EMI SE**  **(dB)** | **SSE**  **(dB cm^3^ g^-1^)** | **SSE/t**  **(dB cm^2^ g^-1^)** | **Absorption**  **(SE_A_/SE_T_)** | **Ref** |
| --- | --- | --- | --- | --- | --- | --- | --- |
| Graphene/Fe_3_O_4_ | 200 | 0.77 | 24 | 31.17 | 1038.96 | 63% | 1^11^ |
| MWCNT/MCMB | 150 | 0.26 | 70 | 119.23 | 7948.72 | 63.7% | 2^12^ |
| LIG/Fe_3_O_4_ | 53 | 0.22 | 32.7 | 148.64 | 28044.6 | 60% | 3^13^ |
| Graphene/Fe_3_O_4_ | 200 | / | 24 | / | / | 63% | 4^7^ |
| sulfonated graphene nanosheets (S-GNS) | 300 | / | 34.4 | / | / | 67% | 5^14^ |
| MXene/ANF | 20 | 1.25 | 28 | 22.4 | 11200 | 62.5% | 6^10^ |
| graphene/waterborne polyurethane | 150 | 1.4 | 30 | 21.43 | 1428.57 | 60% | 7^15^ |
| Carbon nanofber/carbon black/  PI flm | 350 | 1.2 | 23.9 | 19.92 | 569.05 | 62.8% | 8^16^ |
| Ag/CNTs/PDMS | 1500 | / | 56 | / | / | 81% | 9^17^ |
| EG film | 100 | / | 33.1 | / | / | 72% | 10^18^ |
| CF/RGO/Ni | 3300 | / | 61.3 | / | / | 88% | 11^19^ |
| C-MXene@PI foam | 500 | 0.041 | 43.77 | 1065.85 | 21317.07 | / | 12^20^ |
| CNT/graphene/PI foam | 2000 | 0.02 | 28.2 | 1410 | 7050 | / | 13^21^ |
| Graphene/PS | 2500 | 1.04 | 45.1 | 43.37 | 173.46 | / | 14^22^ |
| LIG | 71 | 0.26 | 25.2 | 102.44 | 14428.03 | 66% | This  work |
| double-side LIG | 164 | 0.26 | 36.1 | 146.75 | 8948.05 | 71% |  |
| (F-N-0.7) | 70 | 0.26 | 36.1 | 146.34 | 20905.92 | 75% |  |
| double-side rGO/LIG@NiFe_2_O_4_  (F-N-0.7) | 166 | 0.26 | 50.9 | 206.91 | 12464.49 | 73% |  |

**Reference**

1. Gao, W., Alemany, L. B., Ci, L. & Ajayan, P. M. New insights into the structure and reduction of graphite oxide. *Nat. Chem.* **1**, 403-408, (2009).

2. Park, S. *et al.* Aqueous suspension and characterization of chemically modified graphene sheets. *Chem. Mater.* **20**, 6592-6594, (2008).

3. Chen, C. M. *et al.* Annealing a graphene oxide film to produce a free standing high conductive graphene film. *Carbon* **50**, 659-667, (2012).

4. Pang, H. *et al.* Facile synthesis of a hierarchical multi-layered CNT-NiFe_2_O_4_@ MnO_2_ composite with enhanced microwave absorbing performance. *Appl. Surf. Sci.* **581**, 152363, (2022).

5. Yao, Y. *et al.* Magnetic CoFe_2_O_4_-graphene hybrids: facile synthesis, characterization, and catalytic properties. *Ind. Eng. Chem. Res.* **51**, 6044-6051, (2012).

6. Xu, J. L. *et al.* In-situ plantation of Fe_3_O_4_@C nanoparticles on reduced graphene oxide nanosheet as high-performance anode for lithium/sodium-ion batteries. *Appl. Surf. Sci.* **546**, 149163, (2021).

7. Song, W. L. *et al.* Magnetic and conductive graphene papers toward thin layers of effective electromagnetic shielding. *J. Mater. Chem. A* **3**, 2097-2107, (2015).

8. Shahzad, F. *et al.* Electromagnetic interference shielding with 2D transition metal carbides (MXenes). *Science* **353**, 1137-1140, (2016).

9. Lu, S. *et al.* Flexible, mechanically resilient carbon nanotube composite films for high-efficiency electromagnetic interference shielding. *Carbon* **136**, 387-394, (2018).

10. Xie, F. *et al.* Ultrathin MXene/aramid nanofiber composite paper with excellent mechanical properties for efficient electromagnetic interference shielding. *Nanoscale* **11**, 23382-23391, (2019).

11. Xi, J. *et al.* Graphene aerogel films with expansion enhancement effect of high-performance electromagnetic interference shielding. *Carbon* **135**, 44-51, (2018).

12. Chaudhary, A. *et al.* Lightweight and easily foldable MCMB-MWCNTs composite paper with exceptional electromagnetic interference shielding. *ACS Appl. Mater. Interfaces* **8**, 10600-10608, (2016).

13. Yu, W., Peng, Y., Cao, L., Zhao, W. & Liu, X. Free-standing laser-induced graphene films for high-performance electromagnetic interference shielding. *Carbon* **183**, 600-611, (2021).

14. Wei, L. *et al.* π-π stacking interface design for improving the strength and electromagnetic interference shielding of ultrathin and flexible water-borne polymer/sulfonated graphene composites. *Carbon* **149**, 679-692, (2019).

15. Yang, W. *et al.* Flexible and densified graphene/waterborne polyurethane composite film with thermal conducting property for high performance electromagnetic interference shielding. *Nano Res.* **15**, 9926-9935, (2022).

16. Kong, D., Li, J., Guo, A. & Xiao, X. High temperature electromagnetic shielding shape memory polymer composite. *Chem. Eng. J.* **408**, 127365, (2021).

17. Zhang, J. *et al.* Homogeneous silver nanoparticles decorating 3D carbon nanotube sponges as flexible high-performance electromagnetic shielding composite materials. *Carbon* **165**, 404-411, (2020).

18. Liu, Y. *et al.* Utilizing ammonium persulfate assisted expansion to fabricate flexible expanded graphite films with excellent thermal conductivity by introducing wrinkles. *Carbon* **153**, 565-574, (2019).

19. Bian, X. M. *et al.* Construction of three-dimensional graphene interfaces into carbon fiber textiles for increasing deposition of nickel nanoparticles: flexible hierarchical magnetic textile composites for strong electromagnetic shielding. *Nanotechnology* **28**, 045710, (2016).

20. Zeng, Z. H. *et al.* Porous and ultra-flexible crosslinked MXene/polyimide composites for multifunctional electromagnetic interference shielding. *Nano Micro Lett.* **14**, 59, (2022).

21. Wang, Y. Y., Sun, W. J., Yan, D. X., Dai, K. & Li, Z. M. Ultralight carbon nanotube/graphene/polyimide foam with heterogeneous interfaces for efficient electromagnetic interference shielding and electromagnetic wave absorption. *Carbon* **176**, 118-125, (2021).

22. Yan, D. X. *et al.* Structured reduced graphene oxide/polymer composites for ultra-efficient electromagnetic interference shielding. *Adv. Funct. Mater.* **25**, 559-566, (2015).
